# Supplementary material for: Chronological Age Affects MSC Senescence In Vitro—A Systematic Review
Source: Int J Mol Sci. 2021 Jul 26;22(15):7945. doi: 10.3390/ijms22157945 (PMC8348192; doi:10.3390/ijms22157945)
Supplement: Supplementary file 1 [file ijms-22-07945-s001.zip › ijms-1295720-supplementary.pdf]

	Clinical Trial ID								
	**1**	**2**	**3**	**4**	**5**	**6**	**7**	**8**	**9**
Randomisation of administered dose or exposure level	n/a	n/a	n/a	n/a	n/a	n/a	n/a	n/a	n/a
Allocation concealment	n/a	n/a	n/a	n/a	n/a	n/a	n/a	n/a	n/a
Appropriate participant selection for comparison	++	+	++	++	++	++	++	++	++
Accounting for important confounding/modifying variables	-	-	-	+	+	+	+	+	-
Identical experimental conditions across study groups	++	++	++	++	++	++	++	++	++
Blinding of research personnel	-	-	-	-	-	-	-	-	-
Complete outcome data	++	++	++	++	++	++	++	++	++
Confidence in exposure characterisation	++	++	++	++	++	++	++	++	++
Confidence in outcome assessment (incl. assessor blinding)	+	+	+	+	+	++	+	+	+
Complete reporting of measured outcomes	++	++	++	++	++	++	++	++	++
Other potential threats to internal validity (bias)	-	-	-	++	+	+	++	+	+

Trial ID:

1 = Slotzing et al. (2008); 2 = Wagner et al. (2009); 3 = Pandey et al. (2011); 4 = Alt (2012); 5 = Siegel (2013); 6 = Choudhery (2014); 7 = Ferretti (2015); 8 = Maredziak (2016); 9 = Liu (2017)
